# Global Lysine Crotonylation Alterations of Host Cell Proteins Caused by *Brucella* Effector BspF

**DOI:** 10.3389/fcimb.2020.603457

**Published:** 2021-01-08

**Authors:** Jinying Zhu, Qiao Dong, Changpeng Dong, Xi Zhang, Huan Zhang, Zeliang Chen

**Affiliations:** Key Laboratory of Zoonotic of Liaoning Province, College of Animal Science and Veterinary Medicine, Shenyang Agricultural University, Shenyang, China

**Keywords:** *Brucella*, T4SS, effector, BspF, lysine crotonylation, crotonyltransferase

## Abstract

In *Brucella* spp., the type IV secretion system (T4SS) is essential for bacterial intracellular survival and inhibition of the host innate immune response. The *Brucella* T4SS secretes 15 different effectors to escape host immunity and promote intracellular replication. Among them, BspF has a GNAT-family acetyltransferase domain, implying its acetyltransferase activity. We confirmed that BspF has acetyltransferase activity (data not shown) and de-crotonyltransferase activity. However, BspF overexpressed in HEK-293T cells can also enhance octamer crotonylation *in vitro*. Then we enriched crotonylated proteins and conducted LC-MS to study the crotonylation changes of proteins in HEK-293T cells caused by BspF overexpression. A total of 5,559 crotonylation sites were identified on 1,525 different proteins, of which 331 sites on 265 proteins were significantly changed. We found that Rab9A and RAP1B in proteomics data have a great impact on *Brucella* survival, so we speculate that BspF may influence the function of host proteins by altering crotonylation, thereby promoting the intracellular propagation of *Brucella*.

## Introduction

Post-translational modification (PTM) is a process in which one or more amino acid residues are covalently modified during or after protein translation, and it can be used to aspect of regulate cellular physiology (Mann and Jensen, 2003; Zhang et al., 2011). Different kinds of modifications can change the hydrophobicity, function, and structure of the proteins, as well as their electron-richness and nucleophilic properties. Modifications can enable proteins to control physiological processes, participate in signaling pathways, and even affect immune functions (Liu et al., 2016). Lysine residues on proteins may be methylated, acetylated, biotinylated, ubiquitinated, crotonylated, propionylated, and butyrylated (Wisniewski et al., 2008; Dada et al., 2017). PTMs such as ubiquitination, acetylation, and phosphorylation can regulate the downstream signaling pathway of pathogen recognition by targeting innate sensors, thus affecting the human immune response (Liu et al., 2016). For instance, viral infection can induce C-terminal phosphorylation of interferon regulatory factor 3 (IRF-3), a member of the interferon regulatory factor family, to form a dimer and shift to the nucleus, where it combines with other transcription factors such as CBP/p300 to induce the expression of IFN-α/β and IFN-stimulated genes (Tanaka and Chen, 2012; Cui et al., 2014). Furthermore, phosphorylation of TIR domain-containing adaptor protein inducing interferon-β (TRIF) is required for IRF3 recruitment, which indicates that protein phosphorylation plays an important role in the activation of antiviral innate immune signals (Liu et al., 2015; Liu et al., 2016). In addition, the nuclear factor κB (NF-κB) transcription factor family regulates the expression of a large variety of genes that activate signaling pathways associated with autoimmunity, chronic inflammation, and various cancers (Napetschnig and Wu, 2013). In the TLR pathway, activation of NF-κB and IRF3 requires K63-linked polyubiquitination of TRAF6, TAB2/3, NEMO, and TRAF3, while K48-linked polyubiquitination induces proteasome degradation of IkB, thereby causing nuclear transport of NF-κB (Tokunaga et al., 2009; Napetschnig and Wu, 2013; Liu et al., 2016). Furthermore, TLR stimulation induces acetylation of MAPK phosphatase 1 (MKP1), which increases the interaction between MKP1 and p38, and inhibits the MAPK signaling pathway, which means acetylation may be a negative regulator of innate immune pathways (Cao et al., 2008; Liu et al., 2016).

Lysine crotonylation (Kcr) is a new type of histone modification that was first reported in 2011 by Tan et al., who found that crotonyl enrichment on sex chromosomes promotes gene expression to maintain the activity of haploid cells (Tan et al., 2011; Montellier et al., 2012). Crotonylation also promotes gene expression in response to acute kidney injury (Ruiz-Andres et al., 2016). The transcriptional co-activator p300 has both histone acetyltransferase (HAT) activity and histone crotonyltransferase (HCT) activity, and p300-catalyzed histone crotonylation directly stimulates transcription to a greater degree than does p300-catalyzed histone acetylation (Sabari et al., 2018). Crotonylation is reported on both histone and non-histone proteins. In a study by Xu et al., lysine-crotonylated peptide from human lung adenocarcinoma cell line H1299 was trypsinized and isolated using an anti-crotonylated lysine antibody, and high-resolution liquid chromatography-tandem MS (LC-MS/MS) was performed to determine whether non-histone proteins were modified by crotonylation. This was the first study to show that a large number of non-histone proteins are crotonylated (Xu et al., 2017), but the function of non-histone crotonylation remains unclear. Therefore, *in vitro* studies on crotonyltransferases generally use histone octamers as substrates for experiments (Kollenstart et al., 2019).

Brucellosis is caused by gram-negative facultative intracellular bacteria *Brucella*. It is a worldwide zoonotic disease that impacts reproductive systems in animals and can debilitating humans (Byndloss and Tsolis, 2016). A series of non-specific symptoms such as fever, chills, anorexia, and joint pain occur when humans are infected with brucellosis, which has a huge impact on human health (Zhong et al., 2013). *Brucella* can survive and replicate within a membrane-bound compartment inside professional and nonprofessional phagocytic cells (Naroeni and Porte, 2002), phagocytic cells commonly used in *in vitro* infection are RAW264.7 cells, BMDM cells, etc., and non-phagocytic cells include HeLa cells, Vero cells, and the like (Detilleux et al., 1990; Pizarro-Cerda et al., 1998). The *Brucella* type IV secretion system (T4SS) is a crucial virulence factor that plays an important role in mediating intracellular survival and manipulating host immune responses to infection. After *Brucella* invades the host, the T4SS secretes 15 effectors to promote *Brucella* intracellular survival, namely *Brucella* secreted protein (Bsp) A, B, C, E, and F; *Brucella* TIR protein (Btp) A and B; virB-coregulated effector (Vce) A and C; *Brucella* protein effector (BPE) 005, 043, 123, and 275; Rab2 interacting conserved protein A (RicA); and secreted effector protein A (SepA) (Ke et al., 2015; Dehio and Tsolis, 2017). *Brucella* is phagocytosed by phagocytic cells to form *Brucella*-containing vesicles (BCVs) that interact with early and late lysosomes (eBCV). The maturation of eBCV promotes T4SS secretion. The secreted effector proteins interact with the endoplasmic reticulum exit site and obtain endoplasmic reticulum- and Golgi-derived membranes to form rBCVs. Many host proteins are involved in the development of rBCVs. The protein BspF (BAB1_1948) is a *Brucella* T4SS effector and contains a Gcn5-related N-acetyltransferase (GNAT) family acetyltransferase domain. BspF has been found to promote intracellular bacterial growth by modulating host secretory function (Myeni et al., 2013), but so far, thereare few reports on its structure and function.

In our study, we verified that BspF has de-crotonylase activity *in vitro*, but it can promote crotonylation in cells. Therefore, after overexpression of BspF in HEK-293T cells, we performed crotonylation omics analysis, and speculated that BspF could change protein function to promote intracellular parasitism. At present, there are proteomic analyses of outer-membrane protein (OMP) 25, OMP31, Omp2b porin for *Brucella* (Connolly et al., 2006), *Brucella melitensis* (Wagner et al., 2002), and *Brucella suis* (Al Dahouk et al., 2008). Our study might provide new insights for further understanding the effect of crotonylation on cell signal pathway during the infection.

## Materials and methods

### Plasmid Cloning

The full-length BspF gene was amplified from the chromosome of *B. melitensis* by PCR using BspF-F (CCGGAATTCATGGCTGCAAAAC) and BspF-R (CCGCTCGAGTTATTTATGCTCGG) primers. The PCR amplified genes were gel-purified, and the effector gene BspF was cloned into the pCMV-HA vector (HA-BspF) and pEGX-6p-1 vector (GST-BspF).

### Expression and Purification of BspF *In Vitro*

GST-BspF was transformed into *Escherichia coli* strain Rosetta. The Rosetta was cultured in LB (yeast extract 5 g/L, Tryptone 10 g/L, NaCl 10 g/L) 37°C, 200 rpm, for 12 h. Then collected at 4,000 rpm, resuspended in buffer 1 (KH_2_PO_4_ 1.8 mmol/L, Na_2_HPO_4_ 10 mmol/L, NaCl 137 mmol/L, KCl 2.7 mmol/L, DTT 2 mmol/L), sonicated, and centrifuged at 12,000 rpm to take the supernatant. The supernatant was combined with the GST-affinity column, and the protein that did not bind was washed away with buffer 1, and then incubated with PreScission protease to digest the GST tag.

### Cell Culture, Plasmid Transfection, and Overexpression of BspF

HEK-293T and HeLa cells were cultured in Dulbecco’s minimal essential medium (DMEM) containing 10% fetal bovine serum (FBS) (Gemin, USA) at 37°C and 5% CO_2_.

Plate cells were cultured in 10 mL of 10% growth medium for one day prior to transfection, until they reached 70% to 90% confluency. Lipofectamine 2000 reagent (Thermo Fisher Scientific, USA) were diluted in Opti-MEM Medium (Gibco, Thermo Fisher Scientific, USA), and DNA/RNA was diluted in Opti-MEM Medium and incubated for 10 min. Mixed diluted DNA/RNA and diluted Lipofectamine 2000 gently, incubated for 30 min at room temperature, then added the mix to cells. Six hours after transfection, the growth medium was removed from the cells and replaced with 10 ml of 2% FBS maintenance medium.

Test plates were transfected with 16 μg of HA-BspF plasmid, and control plates were transfected with 16 μg of pCMV-HA plasmid. Cells were collected at four time points: 18 h, 24 h, 30 h, and 36 h after transfection. Protein overexpression was assessed by western blotting.

### *In Vitro* Crotonylation Assays

In vitro crotonylation assays (Kollenstart et al., 2019) were performed with 10 µg BspF, 0.5 µM octamers and 300 µM crotonyl-CoA, in reaction buffer (50 mM Tris-Cl, pH 7.5; 100 mM NaCl; 1 mM EDTA, 1 mM DTT) with a final volume of 50 µl. Reactions were performed for 1 h at 37°C, and inhibited by adding 5 × loading buffer and analyzed by immunoblotting.

### Immunoprecipitation of BspF in HEK-293T Cells for Crotonylation Assay

16 μg HA-BspF and pCMV-HA were transfected into HEK293T cells. Cells were collected at 30 h after transfection, and then lysed with RIPA (150 mM NaCl, 50 mM Tris-HCl (pH 7.4), 2 mM Na_2_ EDTA, 10% glycerol, 1% Nonidet P 40 (NP-40) and 0.1% SDS), centrifuged in a 1.5-ml centrifuge tube at 12000 rpm for 10 min at 4°C, and the supernatant was added to add 2 µl anti-HA (MBL Beijing Biotech Co. LTD, China) for 2 h, then add 50 μl of Protein A/G (Santa Cruz, USA), 4°C, overnight incubation. The agarose beads were washed three times with in reaction buffer.

The BspF protein and interaction protein were pull-down by immunoprecipitation and then reacted with 0.5 µM octamers and 300 µM crotonyl-CoA, for 1 h at 37°C. Reactions were inhibited by adding 5 × loading buffer and analyzed by immunoblotting.

### Protein Extraction and Digestion

Samples were sonicated three times on ice using a high intensity ultrasonic processor (Scientz) in lysis buffer (8 M urea, 1% protease inhibitor cocktail, 3 μM TSA, and 50 mM NAM). Cell debris was removed by centrifugation at 12,000 g at 4°C for 10 min, and the supernatant was collected and protein concentration determined using a BCA kit. The protein solution was reduced with 5 mM dithiothreitol for 30 min at 56°C, then alkylated with 11 mM iodoacetamide for 15 min at room temperature in darkness. Finally, the sample was diluted until the urea concentration was less than 2 M. Trypsin was added at a mass ratio of 1:50 (trypsin: protein) and digestion was performed overnight at 37°C, followed by a 4 h digestion with a trypsin: protein mass ratio of 1:100.

### Affinity Enrichment of Crotonyl Peptides

Peptides were dissolved in NETN buffer solution (100 mM NaCl, 1 mM EDTA, 50 mM Tris-HCl, 0.5% NP-40, pH 8.0), and the supernatant was gently shaken and incubated with pre-washed antibody beads (Lot number PTM503, PTM Bio) at 4°C overnight. After incubation, the beads were washed four times with NETN buffer and washed four times with ddH_2_O. The bead-bound peptides were eluted with 0.1% trifluoroacetic acid three times, and the eluate was collected and vacuum-dried. After draining, the resulting peptides were desalted using C18 ZipTips (Millipore) according to the manufacturer’s instructions for LC-MS/MS analysis.

### In-Gel Digestion

BspF-added group and Non-BspF group were performed SDS-PAGE, and then we cut the substrate from the strip for in-gel tryptic digestion. The gel fragments were decolorized in 50 mM NH_4_HCO_3_ in 50% acetonitrile (v/v), and then use 100 μl of 100% acetonitrile to remove the liquid. The gel fragments were rehydrated in 10 m Mdithiothreitol and incubated at 56°C for 60 min, and dehydrated after incubating for 45 min in the dark. Repeated three times, the gel fragments are rehydrated with 10 ng/μl trypsin and will react with 50 mM NH_4_HCO_3_ for 1 h on ice. The excess liquid was removed, and the gel fragments were trypsinized overnight at 37°C. The peptides were extracted with 50% acetonitrile/5% formic acid and then 100% acetonitrile. And then dry to completion and resuspended in 2% acetonitrile/0.1% formic acid.

### LC-MS/MS Analysis

Peptides were dissolved in solvent A (0.1% formic acid) and directly loaded onto a home-made reversed-phase analytical column (15 cm length, 75 μm id) for separation. The gradient comprised an increase in solvent B (0.1% formic acid in 98% acetonitrile) from 6% to 23% over 26 min, 23% to 35% over 8 min, 35% to 80% over 3 min, and a hold at 80% for the last 3 min, all at a constant flow rate of 400 nL/min on an EASY-nLC 1000 UPLC system.

The separated peptides were injected into an NSI ion source for ionization, followed by tandem mass spectrometry (MS/MS) in Q ExactiveTM Plus (Thermo) coupled online to the UPLC. The voltage was 2.0 kV. The primary mass spectrometer scan range was 350 to 1,600 m/z, the scan resolution was 120,000, and the secondary scan resolution was 15,000. The data acquisition mode used a data-dependent scanning program that alternated between one MS scan followed by 20 MS/MS scans with 15.0 s dynamic exclusion. Automatic gain control was set at 5E4.

### Database Search

Secondary mass spectral data were processed using Maxquant (v1.5.2.8). Tandem mass spectra were searched against the SwissProt Human database (20,317 sequences), and the impact of contaminating proteins in the identification results was minimized as much as possible. Trypsin was specified as the cleavage enzyme, allowing up to 4 missing cleavages. The precursor ions mass error tolerance was 20 ppm for the first search and 5 ppm for the main search, and the mass tolerance for fragment ions was set at 0.02 Da. Cysteine alkylation was specified as a fixed modification, and variable modifications were methionine oxidation, N-terminal acetylation, and lysine crotonylation. The FDR for protein identification and PTM identification was set to 1%.

### Protein-Protein Interaction Network

Search for all differentially expressed proteins in the STRING database of version 10.5, set interactions between proteins that only belong to the search data, thereby excluding external candidates, and set a confidence score> 0.7 (high confidence) for protein interaction analysis. The interactive network of STRING is visualized in Cytoscape. In the figure, the ore clustering algorithm of Molecular Clustering Detection (MCODE) is used to analyze densely connected areas. MCODE is part of the plug-in toolkit of the network analysis and visualization software Cytoscape.

### Immunoblotting

Cell lysates were harvested at 30 h and protein concentration was determined. Equivalent quantities of cell lysates were then denatured in 5 × loading buffer and boiled at 100°C for 10 min. Equal amounts of proteins separated by 10% SDS-PAGE were transferred to polyvinylidene fluoride (PVDF) membranes (Merck Millipore, USA). The membranes were then washed in Tris-buffered saline with Tween 20 (TBST), and blocked in TBST containing 5% skimmed milk for 2 h at room temperature. Membranes were incubated overnight at 4°C with antibodies for detecting crotonylation (PTM Bio, China) and β-actin (Beyotime, China), followed by incubation with HRP-conjugated secondary antibody (Beyotime, China) at room temperature for 2 h. Signals were detected with Clarity ECL reagents (Beyotime, China).

### Experiment of *Brucella* Intracellular Survival

HeLa cells in the six-well plate were cultured for 24 h and then infected with *Brucella*. *Brucella* was collected at the bottom of the centrifuge tube at 12,000 rpm, and the bacteria were resuspended in DMEM. Pipette 200 μl of bacterial solution to infect the HeLa cells, in 37°C incubator for 2 h. Discard the DMEM containing bacteria, wash three times with PBS for washing away the uninfected extracellular bacteria in the culture. Add DMEM containing 2% FBS and 200 μg/ml gentamicin in 37°C for 1 h to kill extracellular bacteria. Change to DMEM containing 2% fetal bovine serum and 50 µg/ml gentamicin, and continue to incubate at 37°C for 24 h. Finally, the cells were lysed with 0.1% (v/v) TritonX-100, the lysate was diluted 10^−4^ and then coated on a TSA plate for counting, repeated 3 times.

## Results

### BspF Contains GCN5-related N-Acetyltransferases (GNAT) Domain and Has De-crotonylase Activity *In Vitro*

**Figure 1:**
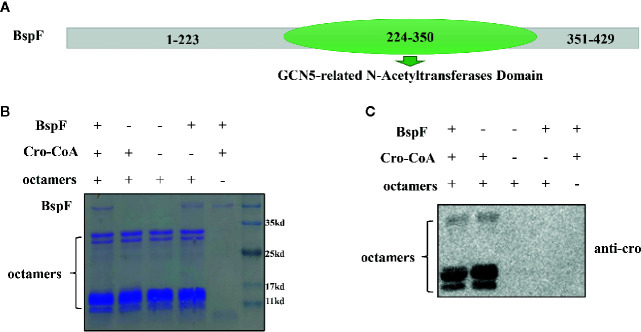
BspF has de-crotonylase activity *in vitro*. **(A)** BspF contains a Gcn5-related N-acetyltransferase (GNAT) family acetyltransferase domain (224-350). **(B, C)** BspF was expressed and purified in *Escherichia coli*, and the *in vitro* BspF shown de-crotonylase activity. **(B)** The SDS-PAGE Coomassie staining of the *in vitro* enzymatic activity experiment reaction system showed that the content of the same components in each system was consistent. **(C)** BspF can attenuate the crotonylation of octamers *in vitro*.

In order to analyze the function of BspF protein, we performed the Conserved Domain Blast of the amino acid sequence of BspF on NCBI. The 224-350th amino acid of BspF is acetyltransferase (GNAT) domain (**Figure 1A**). Most of GNAT-contaning proteins have N-acetyltransferase functions. So we verify the role of BspF in crotonylation *in vitro*. First, we purified BspF, in *Escherichia coli* and tested whether octamers can be crotonylated *in vitro* by acylation transferase assay. Crotonylation was first identified at the lysine site on histones, so we chose octamers as the substrate to verify the crotonyltransferase activity of BspF. We use SDS-PAGE (**Figure 1B**) to ensure that the contents of each group are consistent, and then use anti-crotonyllysine to detect the crotonylation of octamers. As shown in **Figure 1C**, comparing lane 1 and lane 2, the crotonylation on octamers was significantly weakened due to the addition of BspF, so BspF has de-crotonylase activity *in vitro*.

### Mass Spectrometry of Substrate Crotonylation Sites

We verified that BspF has de-crotonylase activity through *in vitro* crotonylation assays, and then we separately identified the two groups of substrates by mass spectrometry (the components in the BspF-added group were 10 μg BspF, 0.5 μM octamers, 300 μM cro-CoA. The components in the Non-BspF group are 0.5 μM octamers, 300 μM cro-CoA) to determine which sites in the substrate have changed crotonylation. We found that octamers in Non-BspF group were crotonylated at K37 of H2A, K21, K47, K117 of H2B, K19, K24 of H3, and K13 of H4. But in BspF-added group, only K47 of H2B and K24 of H3 remain on the octamers with crotonylation (**Table 1**, **Supplementary Table 1**). Indicating BspF has decrotonylase activity *in vitro*.

**Table 1:** Mass spectrometry of substrate.

Sample	Histone octamers	Modification	Modified peptide sequence	Sites*
BspF-added	Histone H2B	Crotonylation	VLk(Cro)QVHPDTGISSK	K 47
	Histone H3	Crotonylation	KQLATk(Cro)AAR	K 24
Non-BspF	Histone H2B	Crotonylation	HAVSEGTk(Cro)AVTK	K 117
	Histone H2B	Crotonylation	VLk(Cro)QVHPDTGISSK	K 47
	Histone H2B	Crotonylation	AVTk(Cro)AQK	K 21
	Histone H4	Crotonylation	GLGk(Cro)GGAK	K 13
	Histone H3	Crotonylation	QLATk(Cro)AAR	K 24
	Histone H3	Crotonylation	k(Cro)QLATK	K 19
	Histone H2A	Crotonylation	k(Cro)GNYAER	K 37

In Non-BspF group, 7 lysine sites of the substrate were crotonylated, and in BspF-added group, only 2 sites on substrate were crotonylated.

Cro stands for crotonylation, *here the site refers to the position of the modification in the entire protein sequence.

### The De-Crotonylase Activity of BspF Is Affected by Its Interacting Proteins in the Intracellular Environment

**Figure 2:**
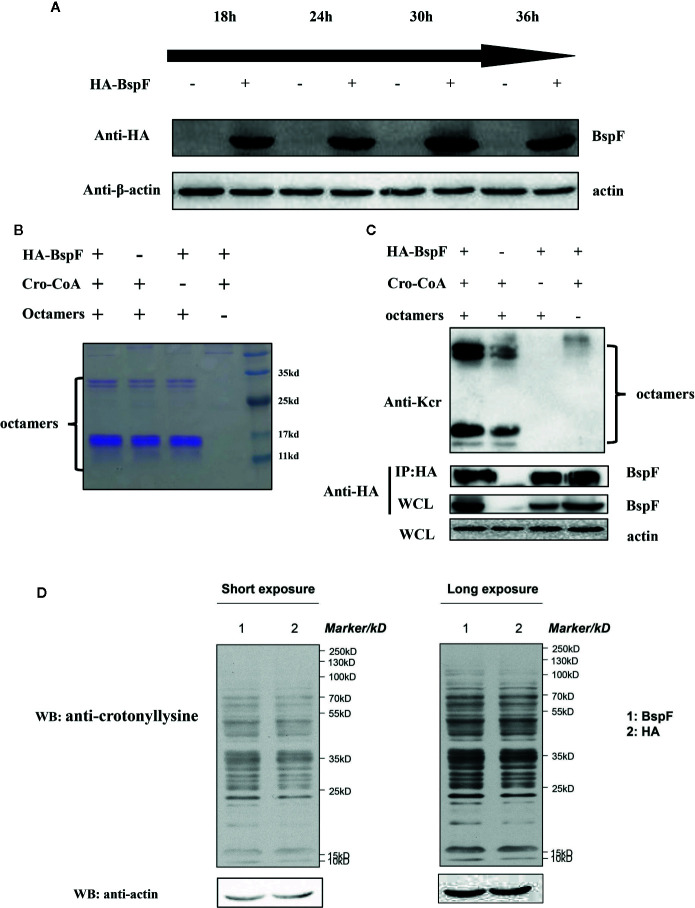
The de-crotonylase activity of BspF overexpressed *in vivo* is affected. **(A)** Overexpression of BspF in HEK-293T cells. At 30 h after transfection, the expression of BspF was highest. **(B, C)** In complex cellular environments, the de-crotonylase activity of BspF is disturbed due to its interacting proteins. **(B)** The SDS-PAGE Coomassie staining of the enzymatic activity experiment reaction system showed that the content of the same components in each system was consistent. **(C)** The protein content of the immunoprecipitation is low, and the expression of BspF cannot be clearly seen in Coomassie staining, so we use western-blot to ensure the expression and uniformity of BspF. Because of interacting partners of BspF, the de-crotonylase activity of BspF is disturbed. **(D)** Western-blot of whole HEK-293T cells lysis with anti-crotonyllysine antibody.

*In vitro* test results showed that BspF has the effect of de-crotonylation, after that we pull-down BspF through immunoprecipitation and incubated with octamers *in vitro*. As shown in **Figure 2A**, BspF expression level increased as transfection progressed, with a peak at 30 h. Then through immunoprecipitation to pull-down BspF and proteins interacted with it. We incubated the pull-down proteins and crotonyl-CoA with octamers at 37°C for 1 h, and we found that BspF and its interacting protein can promote the crotonylation of octamers (**Figures 2B, C**). Subsequently, we determined the presence of crotonylation in HEK-293T cells by immunoblotting, and showed the difference in crotonylation levels between the HA group and the BspF group (**Figure 2D**). We found that BspF has de-crotonylase activity *in vitro* and expressed-BspF in cells may change the crotonylation levels of cell proteins. So we subsequently performed LFQ of lysine crotonylation in BspF-transfected HEK-293T cells.

### LFQ of Lysine Crotonylation in BspF-Transfected HEK-293T Cells

**Figure 3:**
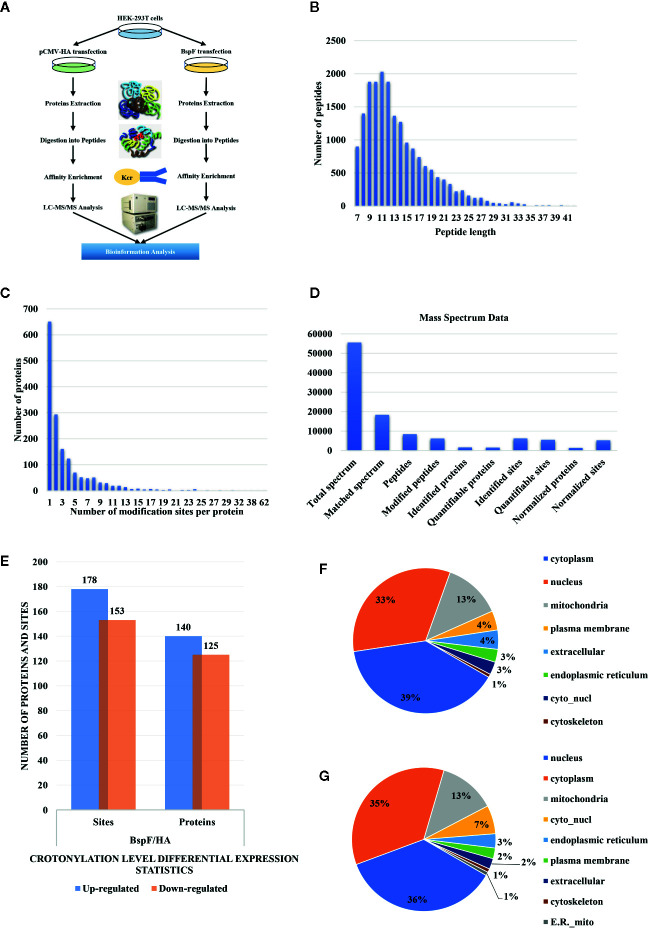
Overview of crotonylation identification in HEK-293T cells. **(A)** Flowchart of the analysis of crotonylation in HEK-293T cells. **(B)** Distribution of tryptic peptide lengths. **(C)** Number of K^Cr^ sites per protein. **(D)** Numbers of proteins and sites that were identified and quantified by MS/MS. **(E)** Differential expression statistics of protein and site modification levels. **(F, G)** Subcellular localization of proteins containing **(F)** up-regulated and **(G)** down-regulated K^Cr^ sites in HEK-293T cells.

We show a flowchart of crotonylation in HEK-293T cells in **Figure 3A**. We analyzed the post-transfection of BspF through immunoblotting with a monoclonal antibody HA. As mentioned above, we chose 30 h post-transfection as the optimal time to perform proteomic analysis.

As shown in **Figure 3B**, the length of the trypsin peptides was predominantly distributed under 25 amino acids. The accuracy of the mass spectrometer is within 5 ppm. In **Figure 3C**, the error distribution between the true value and the theoretical value of the relative molecular weight of all matched peptides. From our crotonylation data (**Figure 3D**), a total of 55,599 secondary spectra were obtained by mass spectrometry analysis. After searching the protein data library, the secondary spectrum of the mass spectrometer obtained an available effective spectrum number of 18,335. A total of 8,433 peptides and 6,209 crotonylated peptides were identified by spectrum analysis segment.

We used label-free quantitative (LFQ) LC-MS/MS proteomics to compare the crotonylation of host proteins in BspF-transfected and HA-transfected cells. In total, 6,245 crotonylation sites were mapped to 1,631 different proteins (**Supplementary Table 2**), with approximately 39.9% of the proteins containing a single putative crotonylated site. Of these, 5,559 crotonylation modification sites on 1,525 different proteins were identified and quantified. Using a quantification ratio of >2 as the upregulation threshold and <0.5 as the downregulation threshold, we found that 178 lysine sites of 140 proteins were upregulated and 153 lysine sites of 125 proteins were downregulated in cells overexpressing BspF (**Figure 3E**).

### Characteristic of Subcellular Locations, Functional Categories, and Crotonylation Motif Related to Host Response

In order to obtain insights into the subcellular locations and biological processes of the proteins exhibiting altered crotonylation in response to BspF overexpression, we performed WoLF PSORT analysis and GO function classification analysis. As shown in **Figure 3F**, the 140 proteins with increased crotonylation were predicted to be located in the cytoplasm (39%), nucleus (33%), mitochondria (13%), plasma membrane (4%), extracellular space (4%), endoplasmic reticulum (3%), cyto_nucl (3%), and cytoskeleton (1%). The 125 proteins with decreased crotonylation (**Figure 3G**) were predicted to be located in the nucleus (36%), cytoplasm (35%), mitochondria (13%), cyto_nucl (7%), endoplasmic reticulum (3%), plasma membrane (2%), extracellular space (2%), cytoskeleton (1%), and ER_mito (1%).

**Figure 4:**
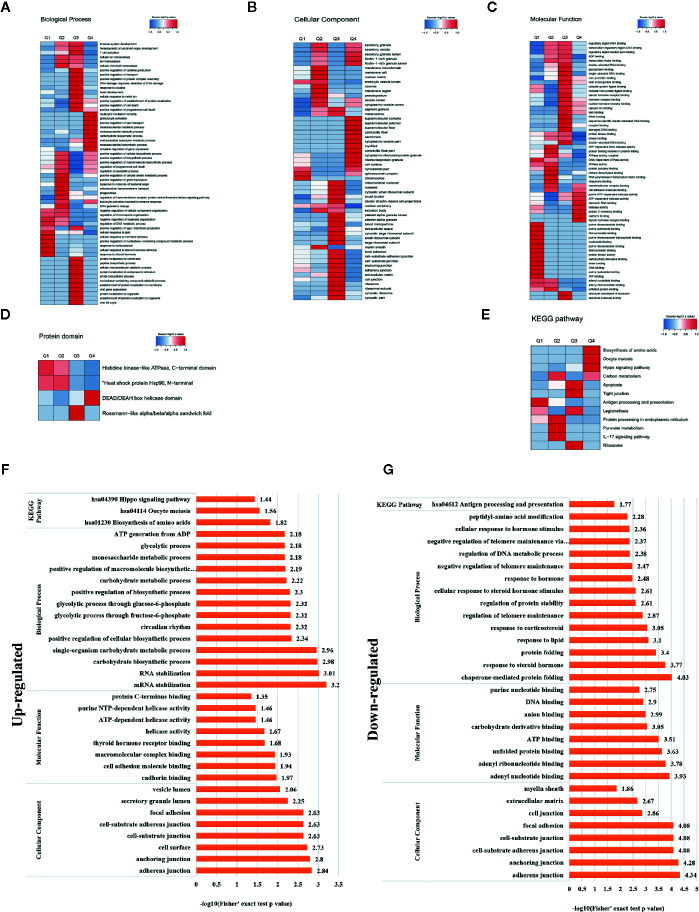
Clustering analysis of identified proteins containing differentially expressed K^Cr^ sites and functional characterization of crotonylated proteins. Modified sites were classified into four classes based on fold change. Heat maps showing the results of a cluster analysis of data from the **(A–C)** GO term, and **(D)** protein domain analyses, and **(E)** KEGG pathway. Functional characterization of crotonylated proteins containing **(F)** up- and **(G)** downregulated K^Cr^ sites at 30 h post-transfection, showing biological processes, cellular components, molecular functions, and KEGG pathways.

A heat map of the clustering analysis (GO, protein domain, KEGG pathway) was generated to present the correlations between differentially crotonylated sites (**Figures 4A–E**). For the KEGG enrichment analysis (**Figure 4B**), differential crotonylated proteins in category Q1, were enriched in “Antigen processing and presentation”. In category Q2, differential crotonylated proteins were enriched in “Carbon metabolism,” “Protein processing in endoplasmic reticulum”, “Pyruvate” and “IL-17 signaling pathway”. Differential crotonylated proteins in Q3, were participated in various cellular activity, including “Apoptosis”, “Tight junction”, “Ribosome” and “Legionellosis”. Finally, in category Q4, differential crotonylated proteins were enriched in “Biosynthesis of amino acids”, “Oocyte meiosis”, and “Hippo signaling pathway”.

In order to gain a deeper understanding of the biological function and process of host protein crotonylation, we compiled detailed annotations of the proteins based on Gene Ontology (GO), protein domain, Kyoto Encyclopedia of Genes and Genomes (KEGG) pathway, and subcellular structure localization. Proteins were classified by GO annotations into three categories: biological process, cellular component, and molecular function (**Figures 4F, G**). All of the differentially crotonylated proteins were predicted to participate in various biological processes such as mRNA stabilization, RNA stabilization, carbohydrate biosynthetic processes, and single-organism carbohydrate metabolic processes. The molecular functions of the crotonylated proteins include cadherin binding and cell adhesion molecule binding, and many of the proteins have helicase activity. We used the KEGG database to predict the pathways in which these crotonylated proteins are involved. As shown in **Figure 4F**, proteins with increased crotonylation are involved in the biosynthesis of amino acids, oocyte meiosis, and the Hippo signaling pathway. And proteins with decreased crotonylation participate in antigen processing and presentation (**Figure 4G**, **Supplementary Table 3**).

We also used soft motif-x to analyze the position-specific frequencies of amino acid residues (**Supplementary Table 4**). A total of 29 motifs were identified, of which four motifs (K^Cr^FXE, AK^Cr^XD, FK^Cr^E, and K^Cr^GXV, where K^Cr^ is the crotonylated lysine and X is any amino acid) showed the highest scores (**Table 2**).

**Table 2:** Crotonylated lysine motifs in HEK-293T cells transfected with BspF.

Motif	Motif score	Foreground		Background		Fold increase
		Matches	Size	Matches	Size	
………FKE………	27.15	64	6011	1614	603638	3.98
……….KL…….K.	24.29	105	5947	3982	602024	2.67
……….KE … K….	22.54	114	5842	4917	598042	2.37
……….KF.E…….	32	84	5728	1607	593125	5.41
.K…….KL………	22.1	86	5644	3649	591518	2.47
……….K.D … K….	25.97	67	5558	2032	587869	3.49
……….KE………	16	625	5491	46789	585837	1.43
….K….KL………	22.71	79	4866	3353	539048	2.61
……V … KD………	22.06	59	4787	2049	535695	3.22
……….KD………	16	416	4685	30259	532475	1.56
………AK.D…….	23.96	43	4728	1171	533646	4.14
……….KF………	16	402	4269	16092	502216	2.94
……….KL………	16	500	3867	41869	486124	1.5
……….KV………	16	431	3367	35501	444255	1.6
……….KM………	16	252	2936	13781	408754	2.55
……….KI………	16	324	2684	28886	394973	1.65
……….KY………	16	215	2360	16105	366087	2.07
………FK……….	16	171	2145	11050	349982	2.52
………YK……….	16	149	1974	9836	338932	2.6
……….KG.V…….	20.64	43	1825	2030	329096	3.82
…….D.K……….	13.05	153	1782	14770	327066	1.9
…….L.K……….	10.98	226	1629	27469	312296	1.58
…….E.K……….	11.51	187	1403	22530	284827	1.69
…….A.K……….	8.98	144	1216	18435	262297	1.68
…….F.K……….	9.5	94	1072	10669	243862	2
……….K.E…….	7.5	117	978	16532	233193	1.69
……….K … F……	6.79	60	861	7274	216661	2.08
…….D.K……….	6.43	80	801	11572	209387	1.81
……….KG………	6.34	106	721	17798	197815	1.63

Totally 29 motifs were identified, the letter K centered in the sequence refers to the crotonylated lysine.

### BspF Causes Crotonylation Changes in Important Protein-Protein Interaction Networks Involved in Transcription and Metabolic Pathway

**Figure 5:**
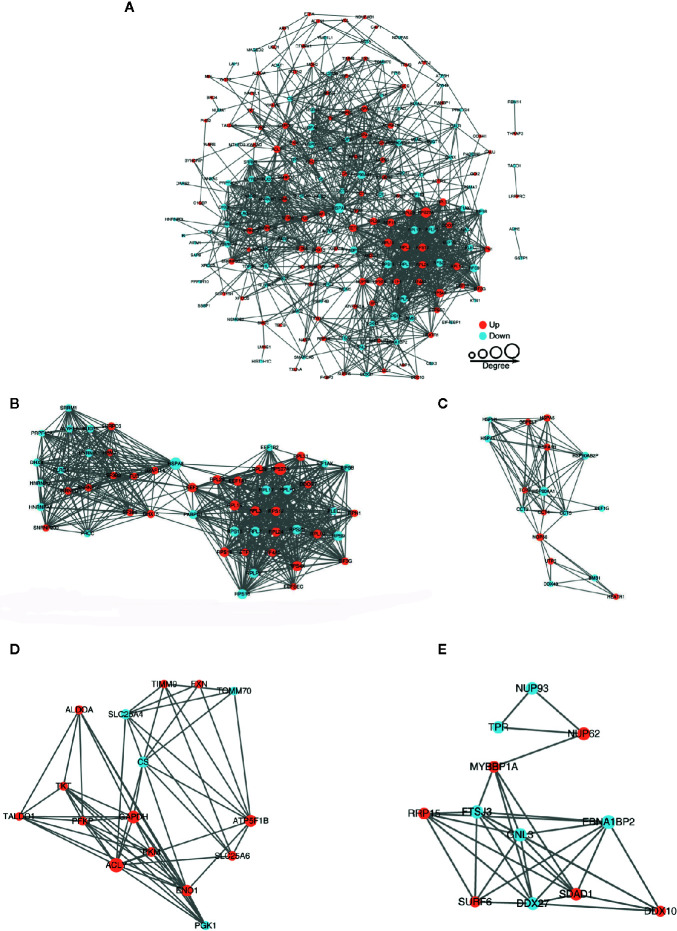
Protein-protein interaction network of **(A)** all differentially crotonylated proteins. The clustering interaction network with MCODE tool **(B)** in Ribosome, **(C)** Endoplasmic reticulum, **(D)** Metabolic pathways and **(E)** RNA transport. Red nodes means up-regulated proteins, blue nodes means down-regulated proteins, the degree of nodes means the number of interacting proteins, the more protein nodes interacting with the protein, the node of this protein is larger.

The protein-protein interaction network consists of individual proteins that interact with each other. The systematic analysis of protein interaction is very important for us to further understand the working principle of protein, response mechanism, energy metabolism and functional connection between proteins. In **Figure 5A**, we screened out all differentially modified proteins and mapped the protein interaction network. We also clustering interaction network with MCODE tool in Ribosome (**Figure 5B**), endoplasmic reticulum (**Figure 5C**), metabolic pathways (**Figure 5D**), and RNA transport (**Figure 5E**). The BspF protein aggregation interaction network shows that the change of intracellular crotonylation level caused by BspF affects the intracellular RNA transport and transcription level, and participates in cell energy metabolism, and may affect the trafficking of *Brucella*-containing vesicles in the cell.

### Rab9A and RAP1B Are Important for *Brucella* Intracellular Survival

**Figure 6:**
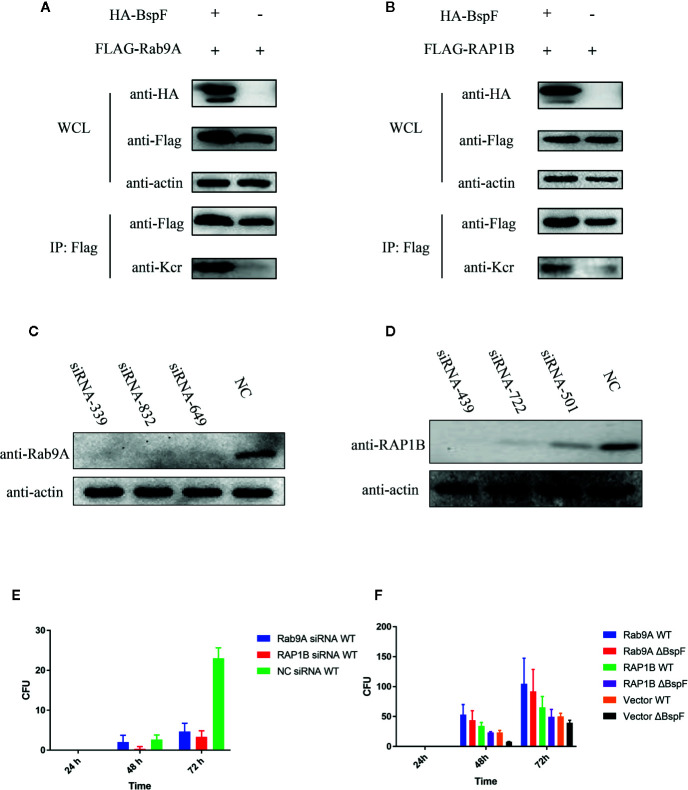
The crotonylated proteins in the crotonylation proteomics data have influence the survival of Brucella. Verification of the accuracy of proteomics data **(A–B) **BspF causing the crotonylation of Rab9A and RAP1B. **(C–D)** Silencing efficiency of siRNA to Rab9A and RAP1B. **(E)** Silencing of Rab9A and RAP1B gene in HeLa cells, the viability of Brucella is greatly reduced. **(F)** The overexpression of Rab9A and RAP1B in the cell enhances the ability of Brucella to survive in the cell.

In our crotonylation proteomics data, Rab9A and RAP1B did not undergo crotonylation in cells, while K4 of Rab9A and K117 of RAP1B were crotonylated with the participation of BspF (**Figures 6A, B**). Subsequently, we silenced Rab9A and RAP1B gene in HeLa cells separately (**Figures 6C, D**), the siRNA-339 silenced Rab9A and the siRNA-439 silenced RAP1B are most efficiently. Then we use *Brucella* wild type (WT) infection the cells after the silencing of Rab9A and RAP1B. As shown as **Figure 6E**, the absence of Rab9A or RAP1B were reduce the intracellular survival of *Brucella*. In addition, we overexpressed Rab9A and RAP1B in HeLa cells and then infected with the WT and ΔBspF of *Brucella*. We found that the increase of Rab9A and RAP1B can promote the survival of *Brucella* (**Figure 6F**). These results all indicate that the proteins in our proteomics data has an effect on the intracellular survival of *Brucella.*

## Discussion

*Brucella* parasitizes the host intracellularly and secretes effector proteins through T4SS to help *Brucella* evade host immunity and promote the reproduction. *Brucella* T4SS effectors BspA, BspB, and BspF inhibit host cell protein secretion and promote *Brucella* intracellular growth and persistence (Myeni et al., 2013). At first, we analyzed the sequence of BspF and performed a conserved domain blast at NCBI. We found that the 224 to 350 amino acids of BspF are acetyltransferase (GNAT) domain. GNAT family N-acetyltransferase catalyzes the transfer of the acetyl group from acetyl coenzyme A donor to a substrate. Due to the structural similarity between the acetyl group and the crotonyl group, many acetyltransferases also have crotonyltransferase activity, such as transcriptional co-activator p300 is a HAT that also participates in histone crotonylation by acting as an HCT (Sabari et al., 2018). We subsequently purified BspF in *Escherichia coli* and verified that BspF has the activity of de-crotonylase *in vitro*. The process of various modifications in organisms is complicated, so we overexpressed BspF in cells, pull-down BspF through immunoprecipitation assay, and then react with octamers *in vitro*. Then we found that the crotonyltransferase of BspF are influenced.

The enzymatic activity of BspF *in vivo* and *in vitro* is completely opposite. We have two hypothesizes. Firstly, the expression of BspF *in vivo* is less than that *in vitro*, which is not enough to attenuate the crotonylation of substrate. Secondly, due to the complexity of the intracellular environment, proteins that interact with BspF through immunoprecipitation are pulled-down, which may contain writers like p300/CBP and MOF, promote substrate crotonylation. Although some bacterial proteins have been identified as having modified enzymatic activities, for example, CobB is de-acetylase in *E. coli*, de-hydroxybutyrylase in *Proteus mirabilis*, and crotonyltransferase in *Streptomyces* (Dong et al., 2019; Sun et al., 2020). As far as we know, we are the first to identify a protein with crotonyltransferase activity in *Brucella* proteins.

On this basis, we over-expressed BspF in HEK-293T cells and performed protein crotonylation proteomics analysis. This study is the first attempt to analyze crotonylation differences between overexpression and non-overexpression of *Brucella* T4SS effector BspF in HEK-293T cells. we used LC-MS/MS to examine changes in protein crotonylation in cells transfected with BspF. After overexpressing BspF in HEK-293T cells, we enriched the crotonylated proteins for proteomic analysis. We identified a total of 6,245 crotonylation sites on 1,631 proteins, of which 5,559 sites on 1,525 proteins contained quantitative information. This was reduced to 5,267 sites on 1,348 proteins following normalization by proteomics. We analyzed the normalized data and introduced a 2-fold-change threshold. Of the quantified crotonylation sites, the crotonylation level on 178 sites were up-regulated in the HA-BspF test group and 153 sites were down-regulated, which shows that the *Brucella* effector change the intracellular crotonylation level.

GO term analysis of proteins with altered K^Cr^ showed that proteins with upregulated crotonylation participate in various biological processes including the stabilization of mRNA and RNA, carbohydrate biosynthetic process, monosaccharide metabolic processes, and glycolytic processes. These results showed that the effector BspF not only affects glycoside metabolism in cells, but also participate in biosynthesis and produces ATP to provide energy. On the other hand, the proteins with downregulated crotonylation are associated with processes involving lipids, corticosteroids, hormones, and hormone stimulation, in addition to chaperone-mediated protein folding and peptidyl amino acid modification. This indicates that BspF affects biochemical reactions in cells, but the specific mechanism of action requires further study.

KEGG analysis showed that proteins with decreased crotonylation were enriched in antigen processing and presentation pathways. Proteins with increased crotonylation were enriched in three pathways: biosynthesis of amino acids, oocyte meiosis, and the Hippo signaling pathway, also known as the Salvador/Warts/Hippo pathway, which is a kinase cascade comprising a series of protein kinases and transcription factors. The Hippo signaling pathway plays a crucial role in adult tissue homeostasis, organ size control, and abnormal regulation of pathways inducing different types of cancers. The core of the Hippo pathway is the kinase cascade of MST1/2 and LATS1/2. Upon activation by upstream signals, MST directly phosphorylates and activates LATS (Callus et al., 2006; Praskova et al., 2008) and the scaffold proteins MOB1 and SAV1. Activated LATS then phosphorylates YAP1 and TAZ, triggering 14-3-3–mediated cytoplasmic retention and protein degradation (Zhao et al., 2010; Kim Y. and Jho, 2018; Kim W. and Jho, 2018). Recent reports indicate that the Hippo signaling pathway participates in the innate immune response, and the Hippo signaling pathway is involved in PD-L1-mediated local immunosuppression in tumor patients. (Taha et al., 2018). In addition, YAP can negatively regulate type I IFN response by inhibiting IRF3 transcriptional activity in cells (Wang et al., 2017; Taha et al., 2018). We speculate that the *Brucella* T4SS effector protein BspF has the potential to alter the modification of intracellular proteins, thereby affecting intracellular Hippo signaling pathway, thus promoting the reproduction of *Brucella*. Hippo signaling pathway is also a part of immune response, which may also be associated with chronically low immunity in patients with brucellosis.

Analyzing the results of LC-MS/MS, we found that the changes of protein crotonylation mainly include two cases. Firstly, the crotonylation level on a lysine site is up-regulated or down-regulated. Secondly, a crotonylation-free lysine site undergoes crotonylation after expression of BspF. Recognition of modifications by “reader” modules constitutes a major mechanism for epigenetic regulation (Xiong et al., 2016). In the first condition, the up-regulated or down-regulated crotonylation on one lysine site will affect reader recognition of crotonyl, thus changing the physiological process of the cell. In the second case, because the crotonylation on one lysine site, the microdomains polarity and the hydrogen bonding of the protein may be changed, resulting in changes of protein’s spatial structure and function.

From our proteomic data, we found that BspF not only caused up- and downregulation of protein crotonylation, but it also catalyzed the modification of some proteins that did not undergo crotonylation at all in the control group, including Rab9A, RAP1B, ATPIF1, USP7, CAND1, NEDD8, and TRIM38. USP7 (also known as HAUSP) is a ubiquitin-specific protease that can inhibit proteasome degradation and maintain protein stability. USP7 is involved in regulating the cell cycle, apoptosis-related factors, and NF-κB transcriptional activity, through stabilization of NF-κB in the nucleus (Colleran et al., 2013; Palazon-Riquelme et al., 2018). BspF-catalyzed crotonylation of the K327 site of USP7 may alter the structure of the protein, thereby causing it to lose its de-ubiquitination function leading to increased ubiquitination of NF-κB and inhibition of NF-κB transcriptional activity. This means that BspF may be responsible for inhibition of the NF-κB signaling pathway and the consequent reduction of inflammatory responses, which provides the necessary conditions for intracellular survival of *Brucella* (Colleran et al., 2013). Rab9A belongs to Ras GTPase superfamily, and participates in trans-Golgi network. Rab9A are essential for parasites and virus (Day et al., 2013; Cardoso et al., 2018). BspF-catalyzed crotonylation of Rab9A K4 site may change the function of protein, affecting the transport of *Brucella* eBCV to the endoplasmic reticulum, thereby promoting the intracellular propagation of *Brucella*.

We first discovered *Brucella* effector has de-crotonylase activit. Through proteomics data, we found that the overexpression of BspF in cells changed intracellular protein crotonylation level. Our follow-up experiments can focus on how BspF interacts with other proteins to increase the level of protein modification in the host. Our proteomics data only provides reference data for the modification level of the host protein of *Brucella* T4SS effector protein BspF during *Brucella* infection, but there are some differences in expression between over expression of BspF and *Brucella* infection. Therefore, these differential proteins require further experiments to verify their modification levels and functions. We speculate that changes in cell proteins crotonylation level affect the function and signaling pathways, and enable multifaceted regulation of the host cell, thereby providing the appropriate conditions for growth and reproduction of *Brucella*. Our research provides new insight for future research on the pathogenic mechanism of *Brucella*, but further research is needed to fully describe the role of protein crotonylation in *Brucella* infection.

## Data Availability Statement

The mass spectrometry proteomics data have been deposited to the ProteomeXchange Consortium *via* the PRIDE partner repository with the dataset identifier PXD021423.

## Author Contributions

ZC and HZ conceived and designed the study. JZ, QD, CD, and XZ participated in data collection and analysis, JZ, HZ, and ZC drafted and revised the manuscript. All authors contributed to the article and approved the submitted version.

## Funding

This work was supported by National Key Research and Development Program Projects of China (2017YFD0500305), National Science Foundation for Young Scientists of China (31702276), National Key Research and Development Program Projects of China (2017YFD0500901), the National Key Program for Infectious Disease of China (2018ZX10101002-002), the State Key Program of National Natural Science of China (U1808202), NSFC International (regional) cooperation and exchange program (31961143024), Major science and technology projects of Inner Mongolia of China.

## Conflict of Interest

The authors declare that the research was conducted in the absence of any commercial or financial relationships that could be construed as a potential conflict of interest.

## Supplementary Material

Supplementary Table 1 Mass spectrometry of histone octamers crotonylation sites.

Supplementary Table 2 Summary and classification of crotonylated proteins and crotonylated sites, including Crotonylation summary, Up-regulated proteins, Down-regulated proteins and Modification distribution.

Supplementary Table 3 Functional classification of differential modification sites correspond to protein, including Biological Process (BP), Cellular Component (CC), Molecular Function (MF) and KEGG pathway (pathway).

Supplementary Table 4 The characteristic sequence of crotonylation site and its enrichment statistics by motif-x.
