# Corrigendum: Global Lysine Crotonylation Alterations of Host Cell Proteins Caused by *Brucella* Effector BspF

**DOI:** 10.3389/fcimb.2021.819711

**Published:** 2021-12-07

**Authors:** Jinying Zhu, Qiao Dong, Changpeng Dong, Xi Zhang, Huan Zhang, Zeliang Chen

**Affiliations:** Key Laboratory of Zoonotic of Liaoning Province, College of Animal Science and Veterinary Medicine, Shenyang Agricultural University, Shenyang, China

**Keywords:** *Brucella*, T4SS, effector, BspF, lysine crotonylation, crotonyltransferase

There is an error in the Correspondence section, as published. The correct order for Correspondence is Huan Zhang (likezhanghuan@aliyun.com) followed by Zeliang Chen (zeliangchen@yahoo.com).

The authors apologize for this error and state that this does not change the scientific conclusions of the article in any way. The original article has been updated.

## Publisher’s Note

All claims expressed in this article are solely those of the authors and do not necessarily represent those of their affiliated organizations, or those of the publisher, the editors and the reviewers. Any product that may be evaluated in this article, or claim that may be made by its manufacturer, is not guaranteed or endorsed by the publisher.

